# Cold Stress in Wheat: Plant Acclimation Responses and Management Strategies

**DOI:** 10.3389/fpls.2021.676884

**Published:** 2021-07-08

**Authors:** Muhammad A. Hassan, Chen Xiang, Muhammad Farooq, Noor Muhammad, Zhang Yan, Xu Hui, Ke Yuanyuan, Attiogbe K. Bruno, Zhang Lele, Li Jincai

**Affiliations:** ^1^School of Agronomy, Anhui Agricultural University, Hefei, China; ^2^Department of Plant Sciences, College of Agricultural and Marine Sciences, Sultan Qaboos University, Muscat, Oman; ^3^Agronomy (Forage Production) Section, Ayub Agricultural Research Institute, Faisalabad, Pakistan; ^4^Jiangsu Collaborative Innovation Centre for Modern Crop Production, Nanjing, China

**Keywords:** wheat, cold stress damage, physiological mechanism, yield, stress management, cold acclimation

## Abstract

Unpredicted variability in temperature is associated with frequent extreme low-temperature events. Wheat is a leading crop in fulfilling global food requirements. Climate-driven temperature extremes influence the vegetative and reproductive growth of wheat, followed by a decrease in yield. This review describes how low temperature induces a series of modifications in the morphophysiological, biochemical, and molecular makeup of wheat and how it is perceived. To cope with these modifications, crop plants turn on their cold-tolerance mechanisms, characterized by accumulating soluble carbohydrates, signaling molecules, and cold tolerance gene expressions. The review also discusses the integrated management approaches to enhance the performance of wheat plants against cold stress. In this review, we propose strategies for improving the adaptive capacity of wheat besides alleviating risks of cold anticipated with climate change.

## Introduction

Climate change is among the core problems of recent times, as it is threatening global food security (FAO, 2020). Uncertain climatic variations poses a severe challenge in fulfilling the future food demands of a growing population (Röder et al., 2014). Extreme temperature events have significantly increased over the past few decades (IPCC, 2014). Persistent cold extremes have been observed in agricultural regions worldwide with varying frequency, intensity, and duration (Kodra et al., 2011; Augspurger, 2013). This situation halts plant growth by causing mechanical injury and metabolic dysfunction through ice crystallization (Yadav, 2010). Most of the wheat-growing areas of the world often undergo low-temperature stress, such as China (Xiao et al., 2018), the United States (Holman et al., 2011), Europe (Trnka et al., 2014), and Australia (Zheng et al., 2015; Crimp et al., 2016). Even though some regions noticed reduced winter duration because of global warming as plant ecologists revealed a paradoxical connection between plant growth and climatic variations, confirming that upsurge in warm climate increased the risk of cold injury to plants (Gu et al., 2008).

Every year, 85% of the wheat sown area in the world is affected by spring frost, and it usually takes place during March and April at the early booting stage (Yue et al., 2016). In the spring season, when wheat canopy temperature falls 0°C or below, severe frost damage occurs (Frederiks et al., 2015; Zheng et al., 2015). Thakur et al. (2010) stated that frequent low-temperature spells during spring cause severe damage to the micro-organelles of the cell, resulting in excessive reactive oxygen species (ROS) and the occurrence of lipid peroxidation. A short span of freezing air during frost stress is disastrous for the vegetative and reproductive growth of plants (Frederiks et al., 2015). Cold conditions disrupt root water uptake, and water inadequacy in the stem results in drought stress (Aroca et al., 2012). This drought condition due to imbalanced water relations causes disturbance in smooth nutrient uptake, decreases the root ion absorption rate, and limits nutrient transport to other plant parts, ultimately resulting in stunted plant growth (Nezhadahmadi et al., 2013).

In a study by Fuller et al. (2007), two wheat cultivars were subjected to cold stress in a freezing chamber with different cold stress treatments for 2 h. Consequently, severe damage to flag leaves and spikes has been observed and the damage increased with temperature decrease. Subsequently, it leads to partial to complete grain yield loss (Fuller et al., 2007). The cold stress also influences the grain number per spike and grain filling rate, leading to a substantial reduction in final wheat production (Thakur et al., 2010). The cold stress-induced yield losses are characterized by a reduced number of productive tillers, spikes, and grains per spike, biologically associated with short stems, lower leaf area, and reduced photosynthetic capacity (Valluru et al., 2012; Li et al., 2015).

The ability of plants to endure cold without damaging their growth cycle is called “cold tolerance” (Liu Y. et al., 2019). The response of plants toward cold stress induction can be classified into four different phases: (i) initial alarming response, (ii) acclimation (the increase in freezing tolerance associated with exposure to low but non-freezing temperatures), (iii) restoration, and (iv) destruction if stress prolongs or severity increases (Larcher, 2003). In addition, after cold stress (i.e., the temperature has risen from cold to optimum), an innate recovery response is activated, providing plant regeneration, an active process after stress cessation, and is vital for further growth and development of plants (Hasanfard et al., 2021). Although the regeneration capability depends on the intensity of stress earlier encountered by the plant (Puijalon et al., 2008), it can be enhanced through exogenous application of certain hormones (i.e., auxin, cytokinin, and strigolactone) (Ikeuchi et al., 2016). The temperate crop plants, including wheat, tend to overcome cold stress through cold acclimation (Theocharis et al., 2012; Li et al., 2014). Cold acclimation of winter wheat can be acquired *via* freeze hardening (the ability of a plant to withstand sub-zero temperature of up to a specific time limit) (Trischuk et al., 2014). This process is carried out through many transcriptional and physiological adjustments, including activation of cold-regulated genes (Zhu et al., 2007; Majláth et al., 2012), downstream regulation of photosynthesis, accumulation of osmoprotectants, and stimulation of antioxidant system (Theocharis et al., 2012).

To maintain yield stability and curtail the negative impact of sudden cold events, adopting proper managerial, and husbandry practices (i.e., sowing method, time, and fertilization) are pretty handy in limiting the risk of frost injury. It is also necessary to develop cold-tolerant wheat cultivars (Limin and Fowler, 2006; Zheng et al., 2015). The cold-defense mechanism of wheat can be improved by implementing integrated multi-disciplinary systems, including screening of cold-tolerant genes through modern gene mapping techniques and developing cold-tolerant cultivars, pre-sowing seed treatments, and applying compatible osmolytes and growth hormones at critical growth stages.

This article reviews the current research findings on how extreme climatic events, particularly cold stress, negatively affect normal wheat growth, development, and yield. It first describes how cold-induced disruptions affect the morphophysiological and metabolic processes, leading to the deterioration of grain quality and lower final grain yield. Following that, it explains how wheat reacts to cold stress by expressing various kinds of adaptive responses. Stress avoidance in wheat (the avoidance of consequences of stress) involves an array of physiological and biochemical modifications (i.e., biosynthesis of compatible osmolytes, protective proteins, alteration in metabolic composition, downregulation of photosynthesis, and ROS detoxification, etc.) that occur simultaneously. Although many studies elucidated cold perception and responsive mechanism in plants, few exist on wheat; this study particularly emphasizes a better understanding of wheat cold perception, with counter-responses concerning futuristic management approaches, as it proposes suitable husbandry practices and multi-disciplinary strategies that can help to anchor the defense of wheat against climatic extremes.

## Responses to Cold Stress

Wheat needs an optimum temperature range for ideal growth and functioning, and any deviation from it will affect the normal growth process (Table 1). Cold stress severely curbs the physiological and biochemical reactions in the plant cell, which results in leaf chlorosis, wilting, and even necrosis of plant cells (Ruelland and Zachowski, 2010). This section briefly discusses how plants perceive low-temperature stress, cold-induced morpho-physiological alterations, and the survival response of the wheat plant.

**Table 1:** Temperature threshold values for various wheat growth stages.

**#**	**Growth phases**	**Min. and Max. (Tolerable Temp. Limit)**	**Optimum Temp. (Ideal Growth Cond.)**	**Optimum duration for growth phases**	**References**
1	Germination and emergence (E)	>4 and <40°C	12–30°C	3.5–10 d (depending on soil moisture)	Spilde, 1989; Mian and Nafziger, 1994; Jame and Cutforth, 2004
2	Floral Initiation (GS1- Prior Vernalization)	−20°C^*^, >20	21–16°C	20 d (Spring) 35 d (Winter)	Evans, 1975
3	Floral Initiation (GS1-Vernalization)	7 and 18°C (Spring) 0 and 7°C (Winter)	4–10°C	5–15 d (Spring) 30–60 d (Winter)	Ahrens and Loomis, 1963; Trione and Metzger, 1970; Evans, 1975
4	Heading to Anthesis (GS2)	>4.5 and <31°C	12°C	100 d (Spring) 130 d (Winter)	Fischer, 1985; Stapper and Fischer, 1990; Acevedo et al., 2009
5	Anthesis to Physiological Maturity (GS3)	>6 and <35.4°C	21°C	140 d (Spring) 170 d (Winter)	Lyons, 1973; Porter and Gawith, 1999

* *Only for winter wheat, while spring wheat shows mild to no response to frost*.

### Morphological Responses and Yield Losses in Wheat

#### Vegetative Phase

When a plant undergoes cold stress, several morphological alterations occur (Equiza et al., 2001); subsequently, root-shoot growth is restricted and productivity is reduced. In winter cereals, low-temperature stress at the vegetative phase cause leaves chlorosis and wilting and ultimately leads to necrosis and inhibited growth (Janowiak et al., 2002). Cold stress severely affects germination and seedling establishment causes delayed germination, poor emergence, reduced plant density, and uneven stand establishment in wheat (Jame and Cutforth, 2004). Winter wheat initially suffers low-temperature stress when tillering begins and whenphotosynthate assimilation and nutrient absorption sites are under development (Rinalducci et al., 2011). Cromey et al. (1998) exposed wheat plants to freezing stress (from 0 to −13°C) in a controlled chamber for 2 h, frost devastation begins at −3°C and complete burning of flag leaf and ears occurred at −7°C; consequently, a substantial reduction in grain yield was observed. Similarly, cold exposure at jointing leads to reduced leaf size, leaf area, and lower shoot biomass (Valluru et al., 2012) and limits the final output (Li et al., 2014). Additionally, applying freezing stress (−8 and −9°C) at the stem elongation stage limits the internode extension, denatures the spikelets, reduces assimilate transport, restricts the dry matter accumulation, and causes a significant reduction in grain yield (Whaley et al., 2004).

Low temperature also affects the root growth of wheat as root growth is an ecologically controlled parameter (Buriro et al., 2011; Kul et al., 2020). Root length is more sensitive to sub-optimal temperature than dry weight. It causes a significant reduction in root branching and root surface area; consequently, normal water and nutrient uptake were disrupted (Hussain et al., 2018). Restricted root surface area inhibits the ability of the plant to explore the water and nutrients resources (Richner et al., 1996).

In summary, cold stress at the initial seedling stage results in delayed emergence and poor stand establishment. Prolonged exposure to cold stress results in stunted growth, diminished root-shoot surface area, leaf chlorosis, and disturbed water and nutrient relations. Such indicators lead to a significant reduction in wheat yield and quality. Additionally, few studies have investigated roots activity with reference to low-temperature stress, which needs to be explored.

#### Reproductive Phase

The reproductive growth phase is more sensitive to cold stress than the vegetative phase in wheat (Thakur et al., 2010). The reproductive growth stage begins with flowering, which continues with floral differentiation (into male and female parts), sporogenesis, pollen grain and embryo development, pollination, fertilization, and, finally, grain development. Plant exposure to cold contact at the reproductive growth phase causes many structural and functional deformities, leading to a reduction in growth and development. Chilling at flowering causes flower shedding, pollen tube deformation (Chakrabarti et al., 2011), pollen sterility, and ovule distortion (Ji et al., 2017), and before anthesis, it lowers down the number of grains and disrupts the grain development (Dolferus et al., 2011; Barton et al., 2014). Such conditions lead to incomplete fruit setting, which reduces the final wheat production (Hussain et al., 2018). Though counterresponse varies at every growth phase, collectively, all responses are not enough to resist net yield loss.

**Figure 1:**
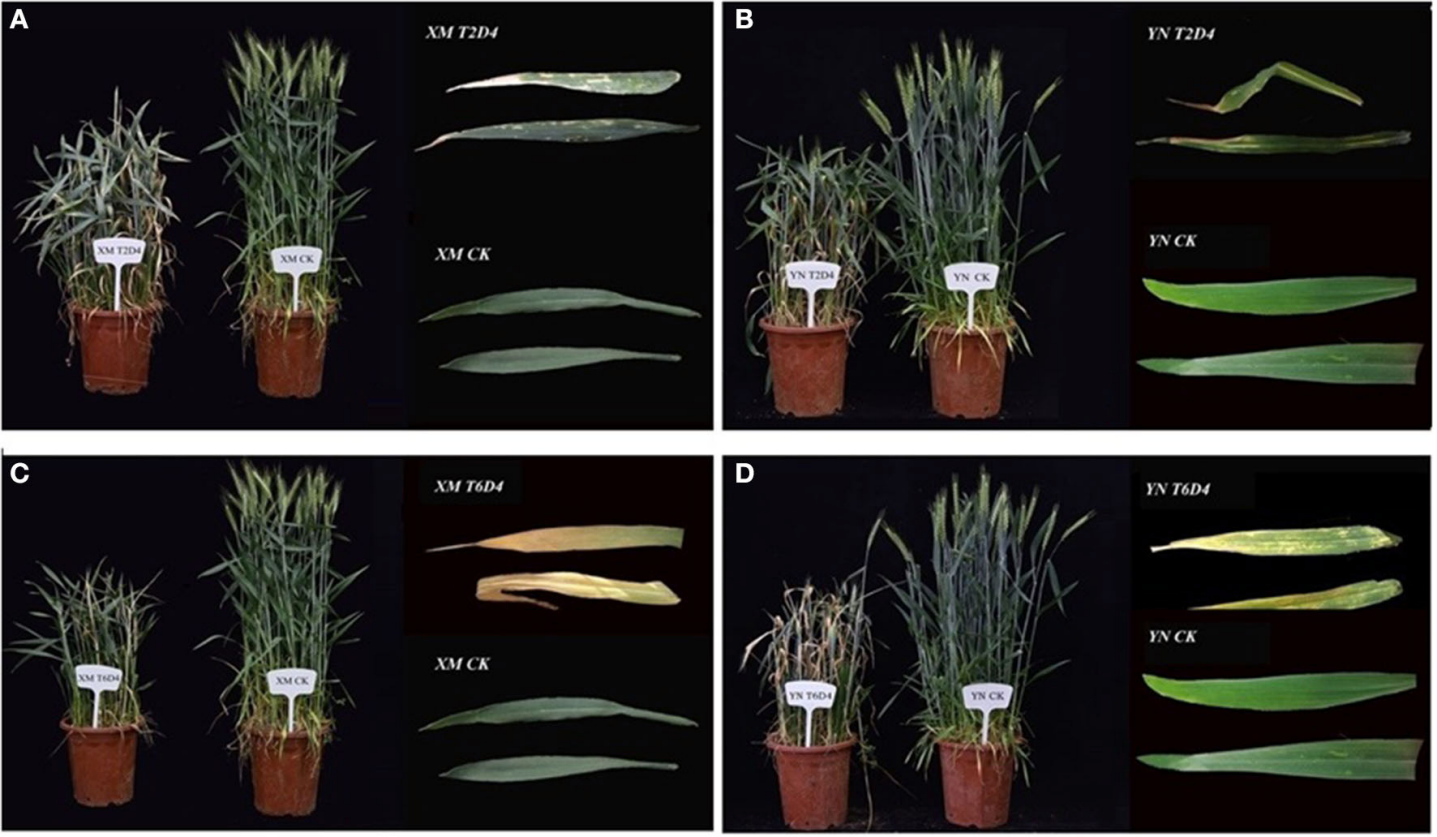
Impacts of low-temperature stress on two winter wheat cultivars, XM and YN (XM: XinMai–Cold Sensitive and YN: YanNong–Cold Tolerant), is shown with the damage induced by cold stress, as compared with control (CK). Wheat cultivars with treatments **(A)** XMT2D4 [T2 = 4°C, D4 = 12 h/3 d], **(B)** YNT2D4 [T2 = 4°C, D4 = 12 h/3 d], **(C)** XMT6D4 [T6 = −4°C, D4 =12 h/3 d], and **(D)** YNT6D4 [T6 =-4°C, D4 = 12 h/3 d) has clearly exhibited the damage induced by cold stress, as compared with control (CK) treatments of XM and YN. Growth Conditions: Experiment grown under field conditions, before the heading stage shifted to the controlled chamber (Humidity: 70%, *Light intensity: 0 μmol·m^−2^·s^−1^) for 3 days (4 h/day, Midnight: 12:00 a.m.−4:00 a.m.) for low-temperature treatments, then shifted back to field conditions. Photos were taken before the flowering stage; extracted leaves are flag/2nd leaf. *In this experiment, in night-time, wheat plants subjected to cold stress, and light intensity set at 0 μmol·m^−2^·s^−1^ because, in field conditions of Huanghuai (China), plants experience late spring cold stress after midnight. (Unpublished: Own Experiment).

**Figure 2:**
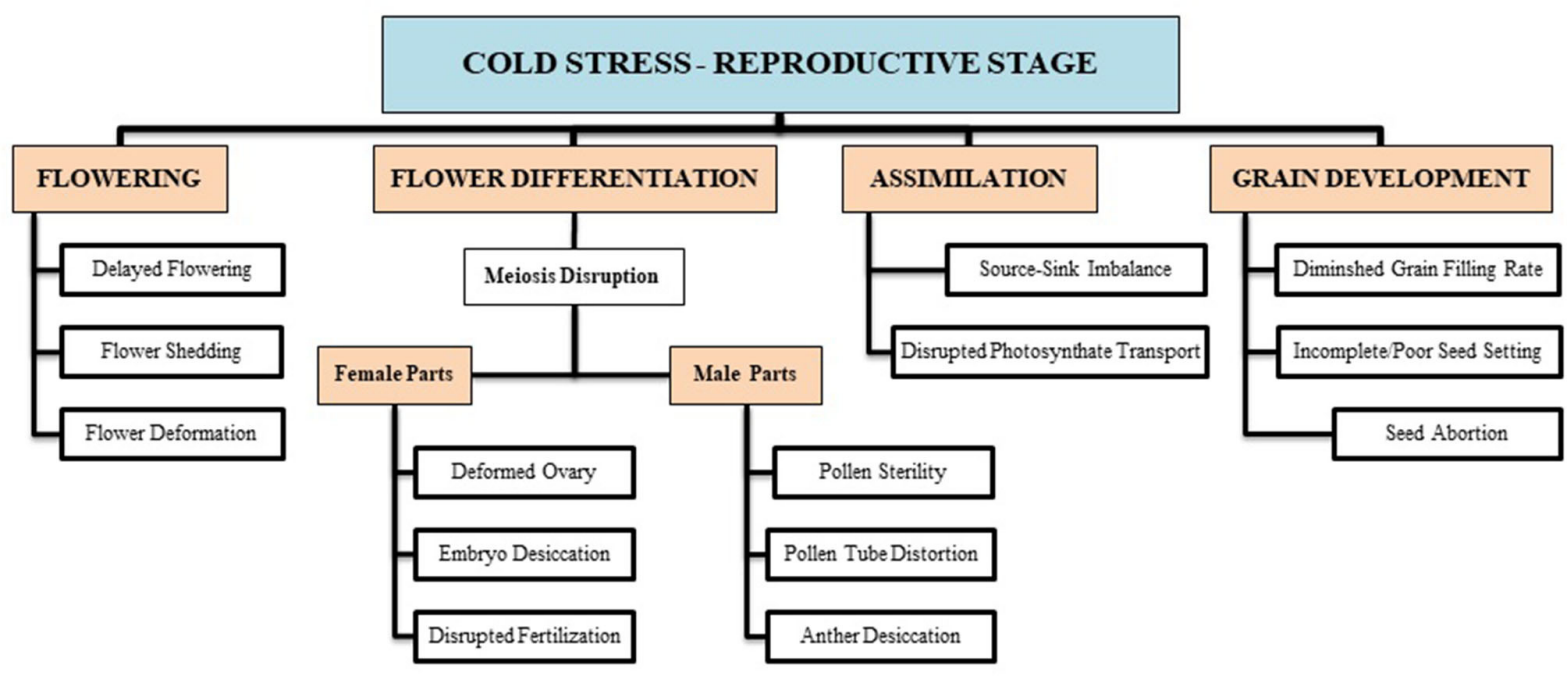
Cold-induced reproductive deformities are briefly illustrated with respect to certain growth stages (i.e., flowering, differentiation, assimilate transport, grain filling, etc.). These growth disruptions result in a substantial decline in final wheat produce.

Imposing chilling and freezing stress at the jointing stage has severely damaged the morphological attributes (such as burned leaf blade, chlorosis, decreased shoot biomass, and denatured spikelets) compared with control (Figure 1). Assimilates accumulation during grain filling is extremely sensitive to suboptimal temperature conditions negatively influencing the grain quality and quantity (Yang and Zhang, 2006). Cold exposure at booting and flowering stages resulted in a considerable reduction in the number of grains per spike; consequently, final grain output diminished to 78% (Subedi et al., 1998). During the reproductive stage, a 1°C decrease in temperature below the threshold level may result in a 10–90% wheat crop damage (Marcellos and Single, 1984; Ji et al., 2017). A field experiment revealed that frost damage of a 5 day span (with a temperature range of 0–4°C) at the stem elongation stage might cause a nearly 15% reduction in the number of spikes and ultimately result in 14% reduction in yield loss (Li et al., 2015). If these frost spells continue, yield losses will be higher (Wu et al., 2014). In conclusion, the wheat crop is more sensitive to cold stress at the reproductive stage, especially the frost spells are disastrous, causing flower shedding, pollen infertility, denatured spikes, and incomplete/poor fruit setting, resulting in significant yield losses (Figure 2).

### Physiological and Biochemical Responses and Wheat Yield

Plant physiological processes, such as photosynthesis and respiration, are more vulnerable to low-temperature stress (Yadav, 2010). Cold stress induces a series of changes in various biological and biochemical processes in the wheat plant cell, including photosynthesis, respiration, water relations, mineral nutrition, and other metabolic activities (Tables 2, 3). In this review, we briefly discussed some of these processes.

**Table 2:** Morphological traits of wheat with respect to their growth stages, influenced by cold stress.

**#**	**Traits**	**Growth stage**	^*^Low Temp. & Duration	**Growth Conditions**	**Cold induced alterations compared to control**	**References**
1	Germination and emergence	Initial seedling stage (Vegetative)	≤ 2°C (>2 d) Control: 30°C	Controlled (Incubator)	Delayed emergence Poor vigor	Jame and Cutforth, 2004
2	Leaf initiation	Seedling growth (Vegetative)	≤ 5°C (12 h to 1 d) Control: 20°C	Controlled (Phytotron)	Leaf initiation ↓ Growth rate ↓ Biomass ↓	Leonardos et al., 2003
3	Ground cover/stand establishment	Tillering (Vegetative)	≤ 0°C (> 5 d)	Field (Frost spells)	Number of tillers ↓ Uneven stand establishment Stem apex killed	Whaley et al., 2004
4	Peduncle development	Stem elongation (Veg. 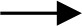 Rep.)	≤ 2 to −9°C (≥ 2 d consecutively)	Field (Frost spells)	Internode extension ↓ Stunted peduncle extension Shoot biomass ↓	Whaley et al., 2004
5	Flag leaf and head emergence	Jointing 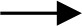 booting (Reproductive)	≤ 0 to −2°C (24–60 h) Control: 8°C	Controlled andOpen-top Chambers	Delayed floret growth Leaf chlorosis and wilting Denatured spikelet	Li et al., 2015; Zhang et al., 2019
6	Flowering, Pollination	Anthesis (Reproductive)	≤ −2 to −6°C (2–6 d) Control: 6°C	Controlled (Phytotron)	Floret abortion Anthers desiccation Flower shedding	Ji et al., 2017
7	Final grain development	Grain filling (Reproductive)	≤ −2 to −6°C (2–6 d) Control: 6°C	Controlled (Phytotron)	Number of grains/spikes ↓ Incomplete fruit setting 1,000-grain weight ↓ Grain Yield ↓	Ji et al., 2017
8	Root growth and development	–	–	–	Surface area ↓ Thickened primary root axis. Lateral branches ↓ Hydraulic conductance ↓ Nutrient uptake ↓	Siddique et al., 2000; Farooq et al., 2009

* *Temperature mentioned here is a minimum recorded field/phytotron temperature during certain growth phases [Here, ↓ indicates a decrease]*.

**Table 3:** Physiological and biochemical traits influenced by cold stress.

**#**	**Processes**	**Effect**	^*^Low Temp. & Duration	**Growth conditions**	**Cold Induced alterations compared to control**	**References**
1	Photosynthesis	Poor photosynthetic activity	≤ 5°C at vegetative phase (1 d) Control: 20°C ≤ 4°C at vegetative phase (1–7 d) Control: 22°C −2 to −6°C at reproductive phase (≥2 d) Control: 6°C	Controlled (Growth Chambers, Phytotrons)	Leaf Area ↓ Leaf water content ↓ Chlorophyll *a,b* synthesis ↓ CO_2_ Assimilation ↓ Quantum efficiency of PSII ↓ Stomatal conductance ↓ Electron transport chain (ETR) ↓ Enzymatic activity ↓ Photo-inhibition Source-sink imbalance	Venzhik et al., 2011; Dahal et al., 2012; Liu L. et al., 2019
2	Respiration	Reduced respiration rate	4^−^12°C at initial vegetative phase (>12 h) Control: 22°C ≤ 5°C at vegetative phase (1 d) Control: 20°C −2 to −6°C at reproductive phase (≥2 d) Control: 6°C	Controlled (Incubator, Phytotrons)	Damaged mitochondrial structure Kinetics of energy flow ↓ Gaseous exchange ↓ Enzymatic activity ↓ ATP production ↓ Metabolism dysfunction Energy reserves ↓	Dahal et al., 2012; Li et al., 2013
3	Nutrient relations	Decreased nutrient uptake and transport			Disturbed soil physio-chemical characteristics Disturbed microbial activity Reduced root surface area, thickened primary root axis and no lateral branching, Hydraulic conductivity ↓ Imbalanced water relations leading drought & reduced phloem activity	Siddique et al., 2000; Farooq et al., 2009

* *Temperature mentioned here is the minimum recorded field/phytotron temperature during certain growth phases, while 20–25°C is the optimum temperature for efficient biochemical functioning Austin, 1990 [Here, ↓ indicates a decrease]*.

#### Cold-Induced Ultra-Structural Impairments

**Figure 3:**
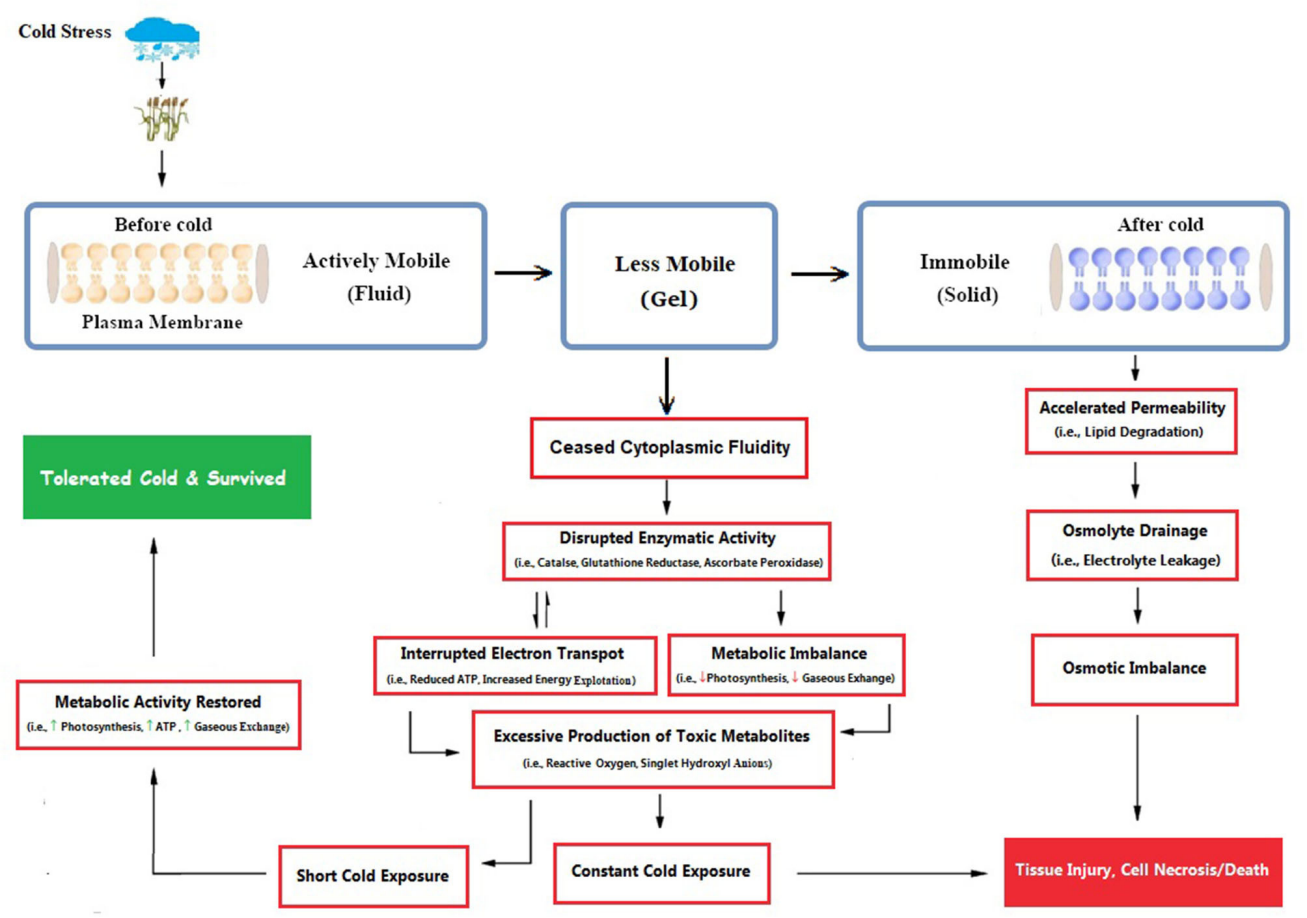
Cold-induced damage is clearly illustrated, as membranes are primarily the first site to get cold damage, followed by a series of osmotic, enzymatic, and metabolic alterations inside the plant cell. Plant cells tolerate shorter cold exposure and restore to normal functioning, but prolonged exposure leads to cell death (Conceived from Seo et al., 2010; Abdel Kader et al., 2011; Theocharis et al., 2012). [Here, ↑ indicates enhanced activity and ↓ shows diminished activity].

Primarily, cellular membranes are the first site of the plant, which is directly affected by cold stress repercussions, leading to other ultra-structural physiological and biochemical changes (Figure 3). Cold stress induces many ultrastructural alterations in cold-sensitive plant species (Pomeroy and Andrews, 1978; Kratsch and Wise, 2000), which causes the imbalance of membrane fluid content and permeability that leads to disturbance to all membrane linked physiological and biochemical processes (Bohn et al., 2007; Los et al., 2013). Most often, these adverse effects are accompanied by structural alterations in the membrane (Bohn et al., 2007), which were subsequently followed by cellular leakage of electrolytes and amino acids, diversion of electron flow toward alternate pathways (Seo et al., 2010), alterations in protoplasmic streaming, and re-distribution of intracellular calcium ions. These severe symptoms are directly correlated with injury to membrane structures of cells and changed lipid composition. Cold-induced alterations in crop plants lead to decreased ATP synthase activity, followed by inhibition of Rubisco regeneration and photophosphorylation (Yordanova and Popova, 2007). Cold-induced photo-inhibition subsequently leads to a reduction in photosynthetic activity (Groom et al., 1990; Oquist et al., 1993). If cold stress remained for a shorter duration, plants could recover their normal state, but such a situation is irreversible under prolonged duration.

#### Photosynthesis

In grains like wheat, photosynthesis, and bio-mass accumulations are the major sources for grain production and vital physiological processes in the crop growth phases; these processes are highly vulnerable to low-temperature stress (Rinalducci et al., 2011; Khan et al., 2017). It has been reported that cold stress causes reductions in final yield, which is associated with a decline in spike number, spike length (Karimi et al., 2011), biomass, leaf area, size, and carbohydrate metabolic reactions. Such morphological and physiological alterations are correlated with reduced photosynthetic efficiency (Theocharis et al., 2012; Valluru et al., 2012). Research findings revealed that imposing low-temperature stress (day:night, 5°C:5°C) at the seedling stage resulted in a 45% decrease in the photosynthetic rate of primary leaves as compared with control (day:night, 20°C:16°C) (Leonardos et al., 2003). Similarly, in another investigation, when wheat seedlings are subjected to low temperature (4°C) in a closed chamber for 7 days, an 18% decrease in photosynthetic activity has been recorded after 5 h. Over-excitation of photosystem II has been observed under cold stress, which triggers energy dissipation through non-radiative reactions (Cvetkovic et al., 2017). The maximum efficiency of Photosystem II decreased by 18% after 1 day exposure to cold (Venzhik et al., 2011). Further, photosynthetic activity in cold-sensitive cultivars is more sensitive to cold stress than cold-tolerant cultivars (Yamori et al., 2009). During the jointing stage, cold exposure inhibits gaseous exchange, thus reducing the quantum efficiency of photosystem II, resulting in decreased photosynthesis that leads to a 5–14% reduction in yield (Li et al., 2015). Flag leaf burning due to freezing stops the photosynthetic activity that resulted in up to 100% yield losses (Rajcan and Swanton, 2001).

Cold-induced photosynthetic inhibition is due to various reasons, i.e., reduced chlorophyll synthesis, poor chloroplast development, diminished efficiency of photosynthetic apparatus, restricted carbohydrates transportation, limited stomatal conductivity, suppressed Rubisco activity during carbon assimilation, disrupted electron transport chain, and decreased energy stock (Bota et al., 2004; Hussain et al., 2018). The chilling conditions instigate drought stress, which reduces molecular oxygen and produces ROS that cause severe damage to photosynthetic apparatus (Basu et al., 2016). During the vegetative stage, cold stress reduced the leaf area, which is considered more critical since it reduces photosynthetic activity, resulting in a source–sink imbalance (Paul and Foyer, 2001; Liu L. et al., 2019; Liu Y. et al., 2019).

Cold stress disrupts the photosynthetic activity at every growth stage, resulting in a reduction in photo-assimilation and assimilate transportation. These conditions lead to significant yield losses.

#### Respiration

The cold stress directly or indirectly induces a series of changes in biological and biochemical functions of the wheat plant, such as decreased respiration rate, reduced enzymatic activity, oxidative stress, and deterioration of seed reserves (Li et al., 2013; Esim et al., 2014). Cold-sensitive plant species, in general, show imbalanced homeostasis of respiration in leaves compared with tolerant species (Yamori et al., 2009). A low respiration rate at the initial seedling stage limits the ATP synthesis; subsequently, the germination process is hindered (Cheplick and Priestley, 1986). The prolonged cold stress period causes severe damage to the mitochondrial structure, slows down the flow of kinetic energy, and disrupts enzymatic activity, ultimately diminishing the respiration rate (Pomeroy and Andrews, 1975; Ikkonen et al., 2020). There are not enough literature studies found in this aspect; particularly for wheat, it is still an under-explored area.

Some studies in soybean reported increased respiration rate under prolonged cold stress; the reason for such increase is irreversible metabolism (dysfunction) and accumulation of oxidized metabolites (Yadegari et al., 2008). Furthermore, it is evident from investigations that chilling triggers the alternative respiratory systems in wheat and maize. Such alternative systems of respiration play a pivotal role in mitigating chilling stress and reducing mitochondrial structural damage (Ribas-Carbo et al., 2000; Feng et al., 2008).

Respiration and photosynthesis are vital processes that define the fate of any plant life. And both physiological processes are prone to cold stress. Structural injury to mitochondria interrupts the energy flow; subsequently, the respiration process is restricted. Such conditions compelled the plant cell to exploit the energy molecules (ATP); energy imbalance disturbed the various biochemical reactions inside the plant cell. Rare studies depict that chilling prompted respiration activity through adopting alternative respiratory pathways and prevented the plant from structural damages.

#### Nutrient Uptake and Transport

Low-temperature stress affects cellular turgidity and instigates drought stress (Yadav, 2010). This drought situation reduces the root hydraulic conductivity, limits the root growth, and dents the leaf turgidity in wheat (Siddique et al., 2000), which causes unavoidable wilting of leaves. Subsequently, water relations, nutrient uptake, carbohydrate metabolism, and translocation of assimilates are severely disrupted (Li et al., 1994). Apart from this, temperature fluctuation trends alter the soil physiochemical properties that disturb the beneficial microbial activity in the soil and influence plant-nutrient relationships (Jezierska-Tys et al., 2012; Massenssini et al., 2015). Further, low temperature slows down root growth and development by reducing root length and biomass. Such a reduction in root volume minimizes the root opportunities to explore new water and nutrient resources; consequently, mineral uptake is severely reduced (Al-Hamdani et al., 1990), resulting in decreased aboveground biomass. Despite the disruption of primary nutrients (NPK), water-deficient conditions also triggered the micronutrient (i.e., Mn, Zn, Fe, Mo, etc.) deficiencies in the plant (Gavito et al., 2001), which otherwise are readily available under well-watered conditions.

In conclusion, there is a direct relationship between nutrient acquisition concerning soil temperature and available soil moisture. It is well-evidenced that chilling and drought directly influence the macro and micronutrient availability, uptake, inflow transport, enzymatic activity, and other metabolic activities in plants. Only few previous studies have been reported on nutrient relations for chilling and drought stress factors; hence, there is a dire need for further investigation.

## Cold Tolerance and Molecular Response

**Figure 4:**
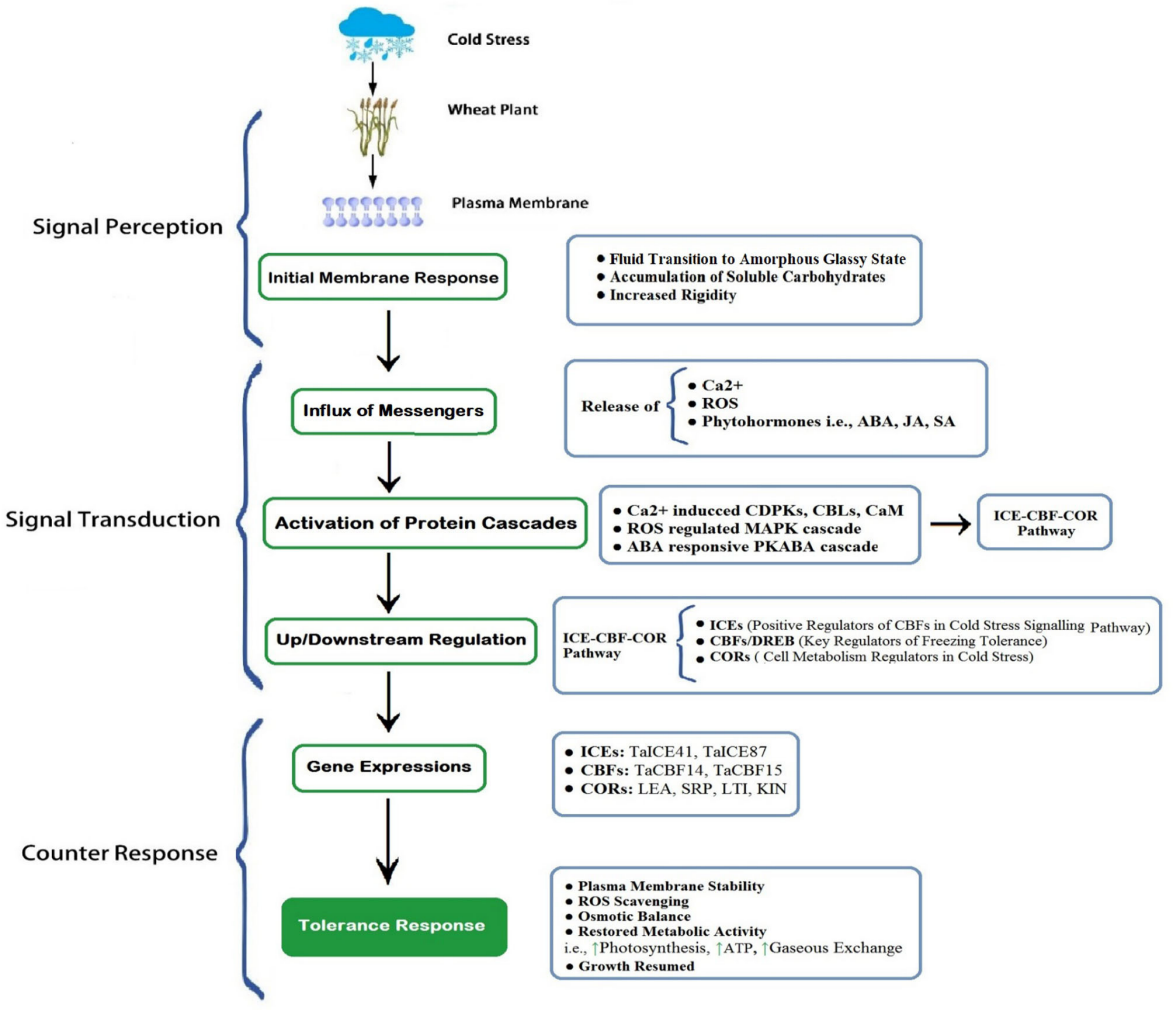
A schematic exhibition of cold perception, transduction, and final counter-response of wheat. The plasma membrane is the first site to perceive cold stress. Membrane rigidity increased with the accumulation of carbohydrates and inside fluid transited to less mobile (gel) or amorphous glassy state. Subsequently, the influx of receptors (Ca^2+^, ROS, Phytohormones), initiation of cascades of protein kinases, and protein cascade-driven up/downstream regulation generate gene expressions to aid cold tolerance (Modified from Guo et al., 2018, 2019). [Here, ↑ indicates enhanced activity]. ROS, Reactive Oxygen Species; ABA, Abscisic Acid; JA, Jasmonic Acid; SA, Salicylic Acid; CDPKs, Ca^2+^-Dependent Protein Kinase; CBL, Calcineurin B-like Proteins; CaM, Calmodulin Proteins; MAPK, Mitogen-Activated Protein Kinase; PKABA, Protein Kinase induced by ABA; ICE, Inducer of CBF Expressions; CBF, Carbon Repeating Binding Factor; COR, Cold Responsive Proteins.

Most of the cereal crops tend to survive and continue their life cycle by developing their tolerance ability under increasing freezing degrees (Dubcovsky and Dvorak, 2007; Thomashow, 2010), through exhibiting a wide range of genetic expressions; such a behavior is termed as cold acclimation (Monroy et al., 2007). Plants having a higher capacity of cold acclimation have more survival chances (McKhann et al., 2008). Generally, winter cereals (wheat) have two types of cultivars: cold sensitive and cold tolerant. Cold tolerant verities have a higher capacity to tolerate sub-optimal cold stress; on the other hand, cold sensitive verities cannot withstand harsh cold conditions. However, winter wheat cultivars having the ability to tolerate suboptimal conditions also requires adequate exposure to non-freezing low temperature, which is crucial for acclimatizing freezing stress (Sung and Amasino, 2005; Majláth et al., 2012). It is revealed in recent developments that the ability of plants to acclimate to the severity of winter gradually decreases with consistent changes in climatic attributes (Dalmannsdottir et al., 2017). Cold acclimation is a complex phenomenon of winter annuals that is accomplished by an extensive range of physiological, biochemical, and molecular changes (Figures 3, 4), which begins with membrane alterations and transforms it into a rigid structure (Theocharis et al., 2012; Takahashi et al., 2013).

### Cold Responsive Protein Expressions

It is stated that the counter action of plants to low-temperature stress is carried out through detection (sensing) of stress followed by signal perception, transduction, and induction of cold-tolerant gene expression (Ganeshan et al., 2008). Plenty of cold responsive genes have been found in wheat (Guo et al., 2019) and are recognized as Dehydrin (DHN), Late Embryogenesis Abundance (LEA), Cold responsive (COR), and Responsive to Abscisic Acid (RBA), among others. These genes are categorized into two parts (Seki et al., 2003): first, those that directly respond to low-temperature stress, i.e., LEA (Liu et al., 2016), and second, those proteins which take part in the regulation of other molecular expressions. In response to cold conditions, these proteins have several other functions as they are also involved in countering other abiotic stresses such as drought and salinity (Seki et al., 2003). During cold tolerance, multiple gene expressions (Arabidopsis, COR and Wheat, WCS) are generated and subsequently initiate the cascade of transcriptional, biochemical, and physiological events vital for cold tolerance in the plant (Kosová et al., 2008). These cold regulatory responses include the release of Ca^2+^, accumulation of osmolytes and reduced water content (Thakur and Nayyar, 2013), ROS scavenging (Larcher, 2003), and carbon metabolic adjustments (Ruelland and Zachowski, 2010). Gene expression, in winter wheat, against cold stress is generated after 1 day (Kurepin et al., 2013), as complete gene expression does necessarily require frequent cold exposure (Ruelland and Zachowski, 2010). The required threshold temperature for initiating this tolerant mechanism varies within two different cultivars of the same species; winter wheat cultivar Norstar and spring wheat cultivar Maintou have rational threshold temperatures of 18 and 8°C, respectively (Fowler, 2008). For the best cold acclimation, the temperature must fall below the threshold level as the acclimation rate is inverse to temperature drop (Chinnusamy et al., 2003).

### Role of ABA in Gene Expression

Abscisic acid induces multiple changes in plant growth, development, and various physiological and molecular processes to cope with stress conditions. It plays a vital role in the tolerance against suboptimal temperature stress by inducing dehydration tolerance gene expressions (Shen et al., 2003). Subjecting to the role of ABA in cold tolerance mechanism (i.e., signaling, perception, and then transduction), it is categorized into two major pathways: ABA-dependent and ABA-independent pathways (Knox et al., 2008; Roychoudhury et al., 2013). ABA-dependent signaling perception of cold stress required ABA activation and vice versa. The gene expressions induced by ABA are carried out through the interaction of various transcriptional factors (such as MYC/MYB, RD22BPI, AREB1, and DREB2A) and their matching cis-elements (such as MYCRS/MYBRS, ABRE, and DRE/CRT), respectively (Tuteja, 2007; Morran et al., 2011).

#### Abscisic Acid-Dependent Pathway

Generally, stress accelerates the biosynthesis of ABA, followed by the closure of stomata and gene expressions (Lee and Luan, 2012). ABA is a primary intracellular receptor that stimulates the activity of secondary messengers, i.e., ROS and Ca^2+^ (Xue-Xuan et al., 2010). The instant signal perception of abiotic stress is transduced *via* the increased ROS and hydrogen peroxide (H_2_O_2_) (Saxena et al., 2016). ROS oxidative surge responded by the release of Ca^2+^ (Rao et al., 2006) that triggers the NADPH oxidase course of action and subsequently proceeds to the accumulation of antioxidant compounds (i.e., H_2_O_2_) (Agarwal et al., 2005). Therefore, Ca2+ is recognized as an essential component in signal transduction. Primarily, three types of Ca^2+^ proteins include CaM (calmodulin), Ca^2+^ dependent kinases, and calcineurin binding proteins. CaM was found to regulate the CBF regulon by binding with the regulatory element of gene promoter and help in cold tolerance (Doherty et al., 2009). Enhanced Ca^2+^ concentration initiates the calcium-regulated protein kinase (CDPKs), which helps in mitigating cold stress. Among 20 CDPKs, 7 responded to various abiotic stresses (Li et al., 2008). ABA-dependent pathway is also dependent on MYB/MYC (myeloblastosis) transcription factors (TFs) (Abe et al., 2003). Among 60 MYB genes of wheat, 15 were characterized as ABA regulated genes (Zhang et al., 2012), such as *TaMYB33*, which plays a part in the production of antioxidants, favoring ROS scavenging, assisting in proline accumulation, and modifying osmotic imbalance (Qin et al., 2012).

#### Abscisic Acid-Independent Pathway

Cereal crops (wheat, barley, and rye) from the Poaceae family contain a large number of DRE or CBF genes, as only wheat contains 25 various kinds of CBF genes (Badawi et al., 2007). COR gene expressions are mainly regulated by CBF TFs (CBF1, CBF2, and CBF3) (Thomashow, 2001). In wheat, the role of CBF has been well-recognized toward many signal perception pathways and enhanced cold tolerance capacity (Morran et al., 2011). Vágújfalvi et al. (2003) found a positive comparative relationship between COR expression and wheat cold acclimation. The COR proteins are labeled as hydrophilic proteins that are considered affiliated with LEA or DHNs (Close, 1997). For instance, in *Arabidopsis thaliana*, overexpression of LEA and wheat cold specific (*WCS*19*)* augments the freeze tolerance capacity (Dong et al., 2002). DHNs or LEA protein groups are highly tolerant to osmotic stress, as cold stress also causes an osmotic imbalance in winter cereals. Hence, their accumulation is equally essential in cold acclimation (Borovskii et al., 2005). The *WCS120* is an impressive cold responsive gene of wheat; it also belongs to the LEA protein family (Fowler, 2001). Along with *WCS120*, other COR genes responsible for cold tolerance include *WCS180, WCS200, WCS66*, and *WCS40* (Sarhan et al., 1997); however, proteins that belong to *WCS120* showed higher transduction in winter cereals and were characterized as best in cold tolerance (Vítámvás and Prášil, 2008).

### Accumulation of Soluble Sugars

Accumulation of soluble sugars is another easier tactic against cold stress. Plants belonging to cereals and grasses families accumulate fructans (fructose polymers derived from sucrose) upon exposure to a cold environment, which plays a stabilizer role in preventing membrane (Livingston et al., 2009). Yokota et al. (2015) also stated a positive correlation between the accumulation of fructans and cold tolerance in wheat plants with varying intensity of cold stress. Carbohydrates accumulation under suboptimal conditions partially support reaching cold tolerance during acclimation (Yoshida et al., 1998; Janská et al., 2010).

Although their specific role at sub-optimal temperature is not fully understood, they are primarily considered as compatible osmoprotectants, ROS scavengers, and signaling compounds. Some plant studies revealed that accumulation of oligosaccharides upon cold exposure anchors the acclimation process (Janská et al., 2010; Theocharis et al., 2012). Fernandez et al. (2010) discussed the role of trehalose (glucose disaccharide) in regulating the cold-tolerant ability, and it is supposed to be involved in starch-accumulation.

Studies on photosynthesis have revealed that some cultivars of winter wheat, compared with spring wheat, sustain carbon assimilation even at cold temperatures, which is associated with increased concentrations of sucrose biosynthetic enzymes (Savitch et al., 1997). Hence, spring wheat lacks in sustaining carbon metabolism under harsh low-temperature conditions. Similarly, another investigation on spring and winter wheat depicted the increased carbohydrate (i.e., sucrose, fructose, and glucose) content in winter wheat, but no upsurge was found in spring wheat, and it also confirmed the role of carbohydrates in the inability of wheat crops to counter low-temperature stresses during spring (Hurry et al., 1995). The role of soluble sugars in responding to the cold signal in plants can be further investigated by advanced molecular techniques to examine how sugar regulates gene expression in a cold environment.

## Management Strategies

Several adaptation strategies facilitate the wheat crops to minimize the negative impacts of low-temperature extremes and are valuable in maintaining global food security. Crop husbandry practices, including the selection of cold-tolerant varieties, nutrient management, appropriate sowing technique and time, seed enhancements, exogenous application of osmoprotectants, and irrigation management, may help improve cold tolerance in wheat. Exogenous application of organic compounds, such as plant polyamines and their derivatives, are considered to be more helpful in enduring both (high and low) temperature extremes (Liu et al., 2007).

### Breeding Advancement Through Implying Multi-Disciplinary Technologies

Breeders are continuously in the quest for developing new cultivars that are more compatible with changing environmental conditions. However, traditional breeding techniques require more than 10 years to develop a new variety. Sudden changes in climatic conditions, especially temperature extremes with drought, are a real challenge for breeders, as it limits the efficiency of new cultivars for a longer time. Along with conventional breeding techniques, it is necessary to implement modern disciplinary techniques in a simultaneous manner, e.g., aid of crop simulation models (i.e., CERES-Wheat Model) is handy in predicting the life duration of the particular cultivar in varying weather extremes and future development of varieties (Koç, 2020). Future assessments will sustainably ensure food security (like availability, accessibility, and continuity) for the growing population (Ray et al., 2013), and it provides enough time for taking decisions against upcoming weather extremes (Archer, 2003). Crop simulation techniques are best in identifying the possible future threats to crop cultivation (Olesen et al., 2007). Furthermore, the addition of modern molecular strategies (like genomics, omics, gene silencing, inducing stress-specific genes, accelerated marker aided selection) and their significance count in the development of high yielding wheat cultivars (Ahmad et al., 2014; Jha et al., 2017). In contrast with traditional breeding tactics, these approaches are more advantageous in improving crop stress responses toward cold and drought conditions (Chaves and Oliveira, 2004).

### Crop Husbandry Practices

Crop husbandry practices may help wheat performance under cold stress. The practices included are as follows: (1) seed enhancements (Farooq et al., 2017), (2) plant seeds of improved wheat varieties following the appropriate planting geometry at optimum sowing time with precision planting (Lamichhane and Soltani, 2020), (3) exogenous application of osmoprotectants, (4) seed inoculation with rhizobacteria (Shirinbayan et al., 2019), (5) nutrient management, and (6) irrigation management.

Positive impacts of seed priming techniques are elucidated in various crop plants under chilling stress (Jisha et al., 2013; Paparella et al., 2015). Primed seeds under low-temperature stress showed enhanced germination rate, improved vigor, and uniform stand establishment, leading to increased crop quality and produce (Paparella et al., 2015). At present, many priming techniques are in practice, such as hormonal priming, biological priming, redox priming, and chemical priming. (Wang et al., 2016). Along with cold stress, these techniques are vital in improving economic output and quality of wheat (Khaliq et al., 2015), maize (Foti et al., 2008), cotton (Casenave and Toselli, 2007), and other field crops.

The foliar application of nutrients and growth hormones is another effective agronomic approach to acclimate the low-temperature stress. Applying phytohormones [i.e., strigolactone, ABA, salicylic acid (SA), jasmonates] accelerate the various protein cascades associated with the expression of cold tolerance genes (Kolaksazov et al., 2013). It also plays a significant role in root–shoot signaling (Wilkinson et al., 2012) and is known to be efficient in minimizing the impact of chilling (Miura and Tada, 2014) and freezing stress (Taşgín et al., 2003).

Additionally, wheat growth sustainability under unfavorable environmental conditions can be achieved by following the agronomic fundamentals, such as optimum planting time, that varies for different regions and cultivars. Optimal sowing time can be determined by certain climatic factors (i.e., air temperature, soil temperature, and moisture content under different climatic conditions). As sowing time depends on climatic variables, it is too difficult to figure out conventional farming approaches under changing climate trends. Therefore, decision support tools of crop modeling are very useful in estimating the optimum sowing time for field crops (Waha et al., 2012). Crop modeling tools can make considerable improvement in evaluating better management strategies for future climatic threats.

Thus, adapting interdisciplinary integrated approaches to tackle the several alarming fronts of climate variability and to ensure the food security of growing populations of the world is urgently needed.

## Conclusion and Prospects

Cold stress causes morphological, physiological, biochemical, and molecular modifications in wheat. Although most winter wheat cultivars tend to tolerate such severe low-temperature extremes, prolonged exposure may result in partial or sometimes complete failure of the final produce. Such an environment/climate induces multiple alterations throughout the crop life cycle, from germination to harvesting. At the early growth stage, suboptimal temperature inhibits the seedling growth and inhibits the developmental process as reduced leaf size, diminished peduncle elongation, and decreased number of tillers and spikes. During the reproductive stage, cold stress results in pollen infertility, floret abortion, reduced fertilization, delayed maturity, and a reduced number of grains. Subsequently, it leads to significant yield losses. Besides, it is well-explained how low-temperature stress influenced physiological and biochemical events, including photosynthesis, respiration, energy imbalance, nutrients, and water relations. Further, to combat cold stress conditions, crop plants exhibit several biochemical and molecular expressions. In addition, the cold-response of wheat can be better regulated by integrating breeding and agronomic approaches, such as phenotypic screening of cold-tolerant genes, pre-sowing seed treatments, and exogenous application of growth hormones.

Due to unexpected climate changes in the last few decades, winter becomes shorter with more severity, damaging winter cereals. There is a greater need to explore and focus on the genetic traits of wheat, due to which it withstands under cold stress and continue their normal growth and development. In this regard, traditional breeding will favor exploiting the wild wheat sources that are more adaptable to natural environmental conditions. It was further improved by identifying diversified genetic traits and mapping through various gene mapping tools, such as QTL mapping and genome-wide association studies (GWAS). In addition, precise and correct gene editing, such as the CRISPR-Cas9 system, will incorporate high yielding cultivars by using genetic engineering techniques. It will also help in ensuring global food security in both quality and quantity aspects.

Apart from this, many other osmolytes (such as glycine betaine) and plant hormones (such as brassinosteroids, ABA, SA, strigolactone) are still not well-exploited and are easier in regulating the plant responses against cold stress. That is why adopting integrated multi-disciplinary approaches to explore these missing links and explore new research horizons is currently needed.

## Author Contributions

MH and CX conceived the concept of the review and prepared an outline of the review. MH, CX, ZY, XH, and ZL compiled the literature and wrote the different sections. KY and AB aided in designing figures and arranging references. MF and NM provided technical assistance and editing support. All authors contributed to the article and approved the submitted version.

## Conflict of Interest

The authors declare that the research was conducted in the absence of any commercial or financial relationships that could be construed as a potential conflict of interest.
